# Supplementary material for: Causal Relationship between Aging and Anorexia Nervosa: A White-Matter-Microstructure-Mediated Mendelian Randomization Analysis
Source: Biomedicines. 2024 Aug 16;12(8):1874. doi: 10.3390/biomedicines12081874 (PMC11351342; doi:10.3390/biomedicines12081874)
Supplement: Supplementary file 1 [file biomedicines-12-01874-s001.zip › Supplementary Figure/Figure S4/Singe SNP plot of MR analysis for TL to ubm-b-1593.pdf]

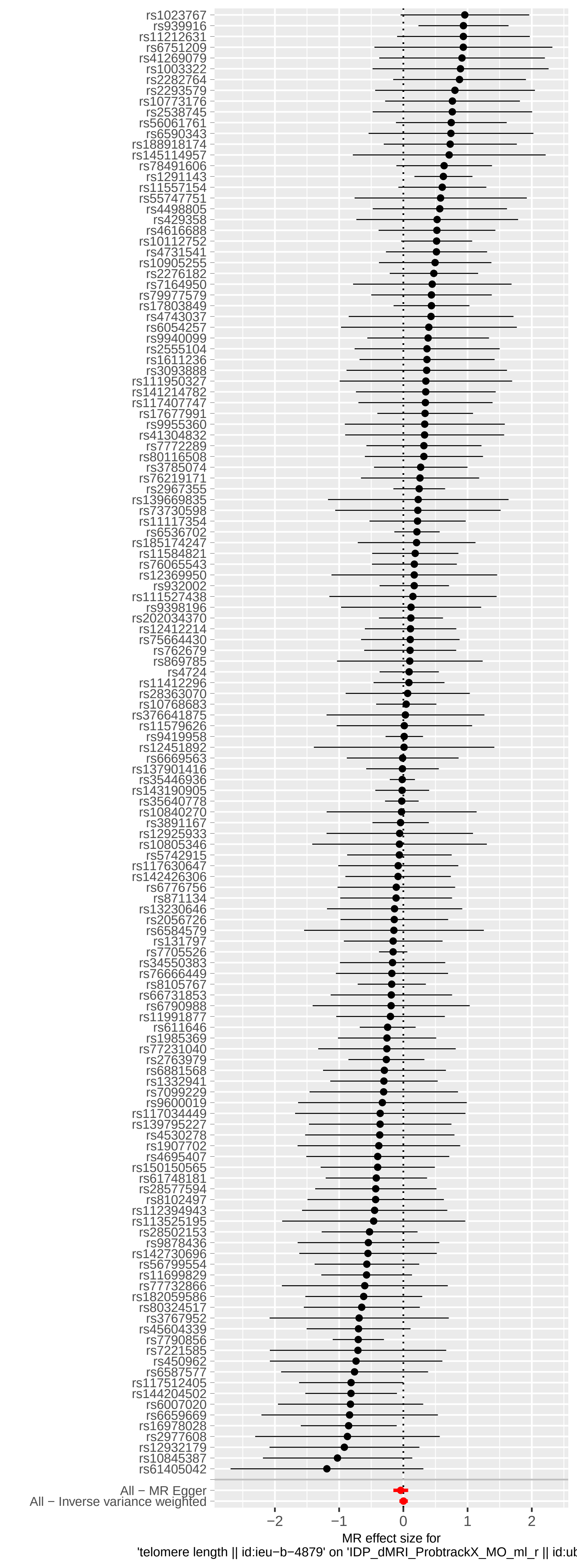
rs1023767
rs939916
rs11212631
rs6751209
rs41269079
rs1003322
rs2282764
rs2293579
rs10773176
rs2538745
rs56061761
rs6590343
rs188918174
rs145114957
rs78491606
rs1291143
rs11557154
rs55747751
rs4498805
rs429358
rs4616688
rs10112752
rs4731541
rs10905255
rs2276182
rs7164950
rs79977579
rs17803849
rs4743037
rs6054257
rs9940099
rs2555104
rs1611236
rs3093888
rs111950327
rs141214782
rs117407747
rs17677991
rs9955360
rs41304832
rs7772289
rs80116508
rs3785074
rs76219171
rs2967355
rs139669835
rs73730598
rs11117354
rs6536702
rs185174247
rs11584821
rs76065543
rs12369950
rs932002
rs111527438
rs9398196
rs202034370
rs12412214
rs75664430
rs762679
rs869785
rs4724
rs11412296
rs28363070
rs10768683
rs376641875
rs11579626
rs9419958
rs12451892
rs6669563
rs137901416
rs35446936
rs143190905
rs35640778
rs10840270
rs3891167
rs12925933
rs10805346
rs5742915
rs117630647
rs142426306
rs6776756
rs871134
rs13230646
rs2056726
rs6584579
rs131797
rs7705526
rs34550383
rs76666449
rs8105767
rs66731853
rs6790988
rs11991877
rs611646
rs1985369
rs77231040
rs2763979
rs6881568
rs1332941
rs7099229
rs9600019
rs117034449
rs139795227
rs4530278
rs1907702
rs4695407
rs150150565
rs61748181
rs28577594
rs8102497
rs112394943
rs113525195
rs28502153
rs9878436
rs142730696
rs56799554
rs11699829
rs77732866
rs182059586
rs80324517
rs3767952
rs45604339
rs7790856
rs7221585
rs450962
rs6587577
rs117512405
rs144204502
rs6007020
rs6659669
rs16978028
rs2977608
rs12932179
rs10845387
rs61405042
All − MR Egger
All − Inverse variance weighted
−2
−1
0
1
2
MR effect size for
'telomere length || id:ieu−b−4879' on 'IDP_dMRI_ProbtrackX_MO_ml_r || id:ub
